# Investigation of *MDM2* Oncogene Copy Number Alterations in Cases of Chronic Lymphocytic Leukemia

**DOI:** 10.4274/tjh.galenos.2018.0218.0270

**Published:** 2019-05-03

**Authors:** Şule Darbaş, Çiğdem Aydın, Ozan Salim, Sibel Berker Karaüzüm

**Affiliations:** 1 Akdeniz University Faculty of Medicine, Department of Medical Biology and Genetics, Antalya, Turkey; 2 Mehmet Akif Ersoy University Bucak School of Health, Department of Nursing, Burdur, Turkey; 3 Akdeniz University Faculty of Medicine, Department of Hematology, Antalya, Turkey

**Keywords:** MDM2, Chronic lymphocytic leukemia, Fluorescence in situ hybridization, P53

## To the Editor,

Chronic lymphocytic leukemia (CLL) is a disease characterized by deposition of malignant monoclonal lymphocytes. Chromosomal abnormalities have been determined in 30%-50% of patients with CLL [1]. The most common chromosomal abnormalities are 13q14 deletion (51%), 11q22.3 deletion (17%-20%), trisomy 12 (15%), 17p13 deletion (7%), 6q23 deletion (7%), and t(14;19) translocation (1%-2%) [2,3].

In CLL patients, overexpression of the *MDM2* gene was shown in earlier studies at protein and RNA levels [4,5,6], and it was aimed to be shown at the DNA level for the first time in this study.

**Figure 1:**
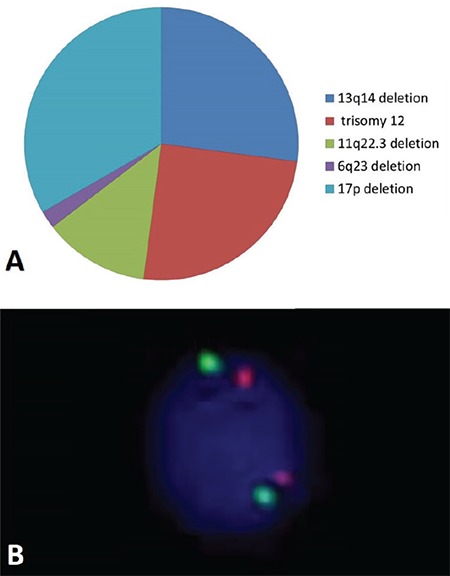
A) Chromosomal abnormalities detected by routine fluorescence in situ hybridization (FISH) analysis of 40 chronic lymphocytic leukemia cases. B) Signal patterns in interphase nuclei of normal FISH results for the *MDM2* gene.

*MDM2* gene amplification was investigated by the fluorescence in situ hybridization (FISH) method in 40 patients with CLL and 20 patients with Ph+ chronic myeloid leukemia as a control group. Informed consent was received. The modified Rai staging system was used for staging our patients. Conventional cytogenetic analysis and FISH analysis using CLL-specific FISH probes for 17p13.1 *(TP53)*, 13q14 *(RB)*, 6q22-q23 *(MYB)*, 11q22.3 *(ATM)*, and chromosome 12 centromere were applied for all patients. The cytogenetic analysis revealed abnormal karyotypes in 3 of 40 patients. 47,XX,inv(9)(p11q13),del(13)(q14),+21[2],46,XY,del (7)(q31),dup(12)(q21q21)[8], and 46,XY,del(20)(q12)[6] karyotypes were observed in these patients. *MDM2* gene amplification could not be detected in either the patient or the control group. FISH analysis results were as follows in CLL cases: deletion of 17p13.1 in 16 cases (40%), 13q14 deletion in 13 cases (32.5%), trisomy 12 in 12 cases (30%), 11q22.3 deletion in 6 cases (15%), and 6q23 deletion in 1 case (2.5%). Frequencies of molecular cytogenetic findings are presented in Figure 1A. Compared to the literature, where the frequency of deletion of 17p13.1 in early-stage CLL was reported between 7% and 10% [7,8], the higher rate observed in 75% of our CLL patients might be due to differences in the methods and probes used, variability of laboratory cut-off values, or the limited number of cases in this study. The clinical implication of having 17p13.1 deletions in CLL cases might be more dependent on the extent of 17p13.1 deletion than the stage of the disease [9]. In the present study, only 4 patients had 17p13.1 deletion in >20 cells. Two of them died because of progressive disease and the other two were lost to follow-up. If evaluated from this perspective, the high level of 17p13.1 deletion was observed in 10% of our cases. It has been observed that patients with 17p13.1 and 11q2.3 deletion have a poor prognosis, and patients with isolated 13q14 deletion were found to have slower progression and longer survival time [2]. We observed that early-stage patients with isolated 13q14 deletion showed slower progression and these patients did not have treatment indications.

MDM2 has pivotal roles in the regulation and stabilization of p53 [10]. In our study, amplification of the *MDM2* gene was not determined in CLL patients, but 30 (75%) of 40 cases were clinically diagnosed as an early stage by the FISH method (Figure 1B). We thought that the absence of *MDM2* gene amplification in our patients might be related to the early stage of the disease. On the other hand, the reason for being unable to observe amplification of the *MDM2* gene in 10 (25%) of 40 patients at advanced stages might be the presence of other abnormalities such as trisomy 12 or deletions of 17p13.1, 11q22.3, and 6q23. We also suggest that reevaluation of *MDM2* gene amplification in patients having a relapse in the future is important for demonstrating the *MDM2*-CLL relationship. In previous studies, *MDM2* overexpression was examined at mRNA and protein levels [4,5,6], but amplification of the *MDM2* gene at DNA level in CLL patients has been examined for the first time in our study.

## References

[1] Glassman AB, Hayes KJ (2005). The value of fluorescence in situ hybridization in the diagnosis and prognosis of chronic lymphocytic leukemia. Cancer Genet Cytogenet.

[2] Döhner H, Stilgenbauer S, Benner A, Leupolt E, Kröber A, Bullinger L, Döhner K, Bentz M, Lichter P (2000). Genomic aberrations and survival in chronic lymphocytic leukemia. N Engl J Med.

[3] Mayr C, Speicher MR, Kofler DM, Buhmann R, Strehl J, Busch R, Hallek M, Wendtner CM (2006). Chromosomal translocations are associated with poor prognosis in chronic lymphocytic leukemia. Blood.

[4] Bueso-Ramos CE, Yang Y, deLeon E, McCown P, Stass SA, Albitar M (1993). The human MDM-2 oncogene is overexpressed in leukemias. Blood.

[5] Haidar MA, El-Hajj H, Bueso-Ramos CE, Manshouri T, Glassman A, Keating MJ, Maher A (1997). Expression profile of MDM-2 proteins in chronic lymphocytic leukemia and their clinical relevance. Am J Hematol.

[6] Winkler D, Schneider C, Kröber A, Pasqualucci L, Lichter P, Döhner H, Stilgenbauer S (2005). Protein expression analysis of chromosome 12 candidate genes in chronic lymphocytic leukemia (CLL). Leukemia.

[7] Yu L, Kim HT, Kasar S, Benien P, Du W, Hoang K, Aw A, Tesar B, Improgo R, Fernandes S, Radhakrishnan S, Klitgaard J, Lee C, Getz G, Setlur SR, Brown JR (2017). Survival of del17p CLL depends on genomic complexity and somatic mutation. Clin Cancer Res.

[8] Hallek M (2017). Chronic lymphocytic leukemia: 2017 update on diagnosis, risk stratification, and treatment. Am J Hematol.

[9] Tam CS, Shanafelt TD, Wierda WG, Abruzzo LV, Van Dyke DL, O’Brien S, Ferrajoli A, Lerner SA, Lynn A, Kay NE, Keating MJ (2009). De novo deletion 17p13. 1 chronic lymphocytic leukemia shows significant clinical heterogeneity: the M. D. Anderson and Mayo Clinic experience. Blood.

[10] Pei D, Zhang Y, Zheng J (2012). Regulation of p53: a collaboration between Mdm2 and Mdmx. Oncotarget.

